# Editorial: Generalizable and explainable artificial intelligence methods for retinal disease analysis: challenges and future trends

**DOI:** 10.3389/fmed.2024.1465369

**Published:** 2024-07-31

**Authors:** Qiang Chen, Theodore Leng, Sijie Niu, Emanuele Trucco

**Affiliations:** ^1^School of Computer Science and Technology, Nanjing University of Science and Technology, Nanjing, China; ^2^School of Information Science and Engineering, University of Jinan, Jinan, China; ^3^Byers Eye Institute at Stanford, Stanford University School of Medicine, Palo Alto, CA, United States; ^4^School of Science and Engineering, University of Dundee, Dundee, United Kingdom

**Keywords:** retinal disease, artificial intelligence technology, biomarkers exploration, generalizable methods, explainable methods, disease prognosis

Retinal diseases encompass a range of conditions that can severely impact vision, including age-related macular degeneration (AMD), diabetic retinopathy (DR), and others. Early diagnosis is crucial for effective treatment and prevention of permanent vision loss. Advances in imaging technology and artificial intelligence (AI) have significantly improved the detection and monitoring of these diseases. AI algorithms can analyze retinal images with high precision, identifying subtle changes that may be missed by the human eye. However, ensuring interpretability and generalizability of AI algorithms remains a critical concern. Continuous research and innovation, coupled with proper safeguards, are essential to enhance diagnostic accuracy and patient care.

AI is revolutionizing medicine, particularly in the analysis of complex diseases such as those affecting the retina. The advent of AI techniques, including deep learning and machine learning, has enabled the automatic extraction of detailed features from retinal images, which significantly aids clinicians in diagnosing and treating retinal conditions. AI models can detect patterns and anomalies that may be missed by the human eye, thereby enhancing diagnostic accuracy and efficiency. Moreover, AI can handle large volumes of data swiftly, making it possible to identify potential biomarkers and predict disease progression. This capability is invaluable in diseases like AMD and DR, where early detection and treatment can drastically improve patient outcomes. By analyzing vast datasets, AI can uncover subtle correlations and trends, contributing to the understanding and management of retinal diseases.

Despite its potential, the use of AI in retinal disease analysis faces significant challenges. One of the primary issues is the *generalizability* of AI models. AI systems trained on specific datasets may not perform well on new, unseen data due to variations in image quality, acquisition parameters, imaging hardware differences, demographic differences, and disease presentation. This limitation can hinder the widespread adoption of AI in clinical settings. To overcome this, researchers must develop AI models that are robust and adaptable to diverse datasets. This requires comprehensive training with varied and representative data, as well as the implementation of advanced techniques like domain adaptation and transfer learning. Ensuring the generalizability of AI models will be crucial for their successful integration into everyday clinical practice.

Another critical challenge is the *explainability* of AI models. Clinicians need to understand how AI systems arrive at their conclusions to trust and effectively use these tools. Black-box models, which do not provide insights into their decision-making processes, are less likely to be accepted by the medical community and the general public.

Developing explainable AI models is essential for their clinical adoption. Explainable AI can provide transparency, allowing clinicians to interpret and validate AI-driven results. Techniques such as saliency maps, which highlight important regions in retinal images, and decision trees, which outline the decision process, can enhance the interpretability of AI models. By making AI systems more transparent, we can bridge the gap between technology and clinical application.

This Research Topic comprises five original research articles which examined several topics including strabismus, age-related macular degeneration, diabetic nephropathy, and diabetic retinopathy. They highlight various aspects of this field of research including bibliometric analysis, synthetic artificial intelligence, federated learning, and disease screening. A summary of these articles is presented as follows.

In recent years, significant advancements have been made in the application of AI to strabismus. Zhou et al. conducted a bibliometric analysis of global publications related to AI in strabismus. Their study, which analyzed 146 relevant publications, found an overall increase in the number of annual publications and citations over the past decade. Keyword analysis indicated that “deep learning” and “machine learning” are likely to be future hotspots. Their findings offer researchers insights into the field's development and guide future research directions.

AMD is one of the leading causes of vision impairment globally. Wang et al. aimed to build a deep learning system for effective detection of AMD. They developed GAN-synthesized fundus photos featuring AMD lesions and evaluated the realism of these images using an objective scale. They used 125,012 real-world non-AMD fundus photos to build this model, then applied a network called StyleGAN2 and the human-in-the-loop (HITL) method to synthesize images with AMD features. Results showed that the synthesized images are realistic and could fool human experts. This research also showed a potential of privacy preservation from synthetic methods.

Gholami et al. present a federated learning (FL) approach for AMD classification in optical coherence tomography (OCT) images. They used residual networks and vision transformer encoders for normal vs. AMD classification, integrating four domain adaptation techniques to address data distribution differences across institutions. Their encouraging experimental results and findings underscore the potential of FL strategies.

Diabetes mellitus is a chronic disease that threatens overall health and wellbeing. Diabetic nephropathy (DN) and DR are two common complications associated with this condition. Liu et al. proposed a deep learning algorithm for early screening of DN patients. They used the Grad-CAM algorithm to identify image regions significantly impacting model output, combining it with attention mechanisms to enhance the model's focus on lesion areas in retinal OCT images. Their highly accurate results show that the proposed model improved the identification of lesion areas.

For the screening of DR, Barakat et al. recognized that AI-based screening programs are gradually outperforming traditional methods. However, Saudi Arabia has not yet established a national DR screening program. The primary aim of the research reported is to assess the awareness and acceptance of AI in DR screening among ophthalmic care professionals in Saudi Arabia. Overall, there was a positive attitude toward AI-based DR screening, while concerns about the job market and data confidentiality were evident.

In summary, this Research Topic focuses on generalizable and explainable insights into the retinal disease analysis methods. Privacy protection and data security are inevitable issues in medical image analysis. Generative methods and federated learning offer viable and promising solutions to these challenges. Early diagnosis of diseases provides a foundation for timely, hence effective, treatment and management. Incorporating interpretable disease regions or biomarkers into the model's decision-making process can enhance the accuracy and reliability of screening results.

## Author contributions

QC: Writing – original draft, Funding acquisition. TL: Writing – review & editing, Funding acquisition. SN: Writing – review & editing. ET: Writing – review & editing.

